# 58-F, a flavanone from *Ophiopogon japonicus*, prevents hepatocyte death by decreasing lysosomal membrane permeability

**DOI:** 10.1038/srep27875

**Published:** 2016-06-16

**Authors:** Xiaofeng Yan, Tingjie Ye, Xudong Hu, Pei Zhao, Xiaoling Wang

**Affiliations:** 1 Department of Biology, school of basic medical science, Shanghai University of Traditional Chinese Medicine, Shanghai 201203, China

## Abstract

Lysosome membrane permeabilization (LMP) has been implicated in cell death. In the present study, we investigated the relationship between cell death and H_2_O_2_-/CCl_4_-induced LMP in hepatocytes *in vitro* and following acute liver injury *in vivo*. The key finding was that H_2_O_2_ triggered LMP by oxidative stress, as evidenced by a suppression of LAMP1 expression, a reduction in LysoTracker Green and AO staining, and the leakage of proton and cathepsin B/D from the lysosome to the cytoplasm, resulting in cell death. CCl_4_ also triggered hepatocyte death by decreasing lysosome LAMP1 expression and by inducing the accumulation of products of peroxidative lipids and oxidized proteins. Furthermore, a novel compound 5,8-dimethoxy-6-methyl-7-hydroxy-3-3(2-hydroxy-4-methoxybenzyl) chroman-4-one (58-F) was extracted from *Ophiopogon japonicus* and served as a potential therapeutic drug. *In vivo* and *in vitro* results showed that 58-F effectively rescued hepatocytes by decreasing LMP and by inducing lysosomal enzyme translocation to the cytosol.

Lysosomes are the cellular recycling centre. They are filled with more than 50 soluble acid hydrolases that mediate the degradation of the luminal cargo or damaged proteins. Acid hydrolases include proteases such as the aspartic cathepsin D and cysteine cathepsins B, C, H, K, L, S and X^1^. The pH of the cytoplasm is usually slightly alkaline (pH 7.2)^2^. There are three highly glycosylated lysosome-associated membrane proteins (LAMPs), called LAMP1, LAMP2 and LAMP3, in lysosome membranes^3^. Carbohydrates constitute 55–65% of the total mass of LAMPs, and the majority of the carbohydrates’ resides in the luminal side of the lysosome. The abundance of LAMP molecules is so high that LAMP molecules form a nearly continuous coating on the inner surface of the lysosomal membrane and serve as a barrier to soluble hydrolases^4^. The lysosomal membrane plays a vital role in the normal function of the lysosome by sequestering the acid hydrolases from the other cytoplasmic components. In addition to the specific lysosome membrane proteins, other proteins also play important roles in lysosomal membrane integrity, such as phosphofurin acidic cluster sorting proteins (PACS), which recruit Bim and Bax to the lysosome to release cathepsin B and induce apoptosis^5^,^6^. The loss of membrane integrity leads to lysosomal membrane permeabilization (LMP), allowing the release of the luminal contents, such as proteases and protons, into the cytosol, resulting in lysosomal acidification inhibition and cell death. LMP and the consequent leakage of the lysosomal contents into the cytosol lead to “lysosomal cell death”, which is mainly catalysed by lysosomal cathepsin proteases. Lysosomal cell death is characterized by necrotic, apoptotic or apoptosis-like features depending on leakage of the lysosomal contents and the cellular context^7^. However, the molecular mechanisms of LMP-induced cell death remain to be elucidated.

Reactive oxygen species (ROS) and their signalling are important factors that regulate a number of processes under physiological conditions. However, oxidative stress caused by the imbalance of ROS production and clearance has been associated with numerous pathological consequences, including necrosis, autophagy, and apoptosis^8^. Irrespective of the aetiology, hepatocytes are usually the first vulnerable target cells in the liver, leading to increased production of ROS and oxidative stress. An increase in oxidative stress also sensitizes hepatocytes to subsequent necrosis and/or apoptosis and ultimately results in a massive loss of mature hepatocytes with subsequent inflammation, fibrosis, cirrhosis, and even hepatocellular carcinoma^9^. The mechanisms that mediate LMP-induced hepatocyte death caused by ROS remain to be elucidated.

5,8-Dimethoxy-6-methyl-7-hydroxy-3-3(2-hydroxy-4-methoxybenzyl)chroman-4-one (58-F) is a flavanone and a novel compound extracted from the traditional Chinese herb *Ophiopogon japonicas,* which is widely distributed and used clinically in mainland China^10^,^11^.

The present study used the CCl_4_-induced mouse liver injury model and the H_2_O_2_-induced BNL CL.2 hepatocyte cell line injury model to test the hypothesis *in vitro* and *in vivo*, respectively, that ROS-induced LMP leads to leakage of the lysosomal contents into the cytosol and triggers cell death in hepatocytes and that the compound 58-F may play a protective role in this process. Our findings demonstrate that LMP and the consequent leakage of the lysosomal contents into the cytosol lead to cell death and that 58-F protected hepatocytes by blocking LMP. The results support our hypothesis, identifying LMP as the primary cause for ROS-induced death of hepatocytes and supporting the protective role of 58-F.

## Results

### 58-F Attenuates Lysosomal Damage after CCl_4_-induced Acute Liver Injury

**Figure 1:**
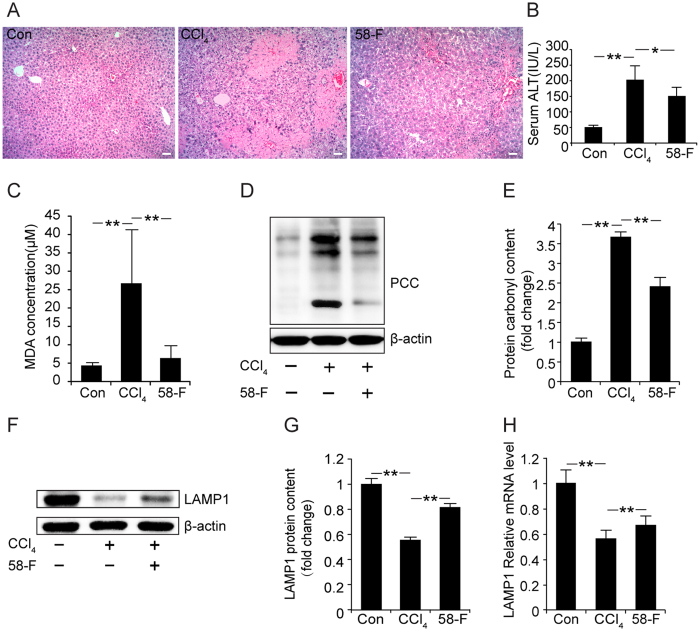
Effect of 58-F on CCl_4_-induced liver injury. (**A**) HE staining is shown (scale bar = 100 μm). con: control group (n = 6), CCl_4_: CCl_4_ injection group (n = 9), 58-F: CCl_4_ injection and 58-F administration group (n = 9). (**B**) Serum ALT activity in mice is shown. (**C**) MDA content of liver tissues in mice was determined. (**D**) Western blot analysis was performed to determine the PCC content in the liver tissues in mice. (**E**) The PCC expression measured in (**D**) was quantified. (**F)** Western blot analysis was performed to determine the LAMP1 content of liver tissues in mice. (**G**) The LAMP1 expression measured in (**F**) was quantified. (**H**) The LAMP1 mRNA levels from liver tissues in mice were quantified by qRT-PCR. mRNA levels were normalized to those of 18S rRNA. All data are presented as the mean ± SD. Statistical analysis was performed using Student’s *t* test. (*p < 0.05, **p < 0.01).

Haematoxylin and eosin (H&E) staining revealed normal hepatic architecture within the clear hepatic lobule and no dead hepatocytes. However, a large area of dead hepatocytes was detected in the central vein area of the CCl_4_-treated mice; the number of dead hepatocytes was significantly decreased following 58-F pretreatment (Fig. 1A). ALT is an enzyme in the cytoplasm of normal hepatocytes. When the integrity of the cell membrane is damaged, ALT is released from the hepatocytes into the serum. Therefore, ALT can be used an index of liver injury in the clinic and for *in vivo* studies^12^. The administration of CCl_4_ to mice resulted in severe hepatotoxicity, reflected by a significantly elevated ALT level (p < 0.01). Pretreatment with 58-F prevented CCl_4_-mediated toxicity by decreasing the ALT level (p < 0.05) (Fig. 1B).

Malondialdehyde (MDA) is a final product of lipid peroxidation, so its level can be used as a biomarker of lipid peroxidation in the liver^13^. The protein carbonyl content (PCC) is used to evaluate the total level of protein oxidation. The MDA level and PCC following CCl_4_ treatment both increased significantly (p < 0.01). Pretreatment with 58-F reduced both MDA levels and the PCC (p < 0.01) (Fig. 1C–E).

LAMP1, used as a lysosomal marker, is expressed largely in the endosome-lysosome membranes of cells^3^. Both LAMP1 mRNA and protein levels decreased significantly after CCl_4_ treatment (p < 0.01), and 58-F pretreatment increased both mRNA and protein levels (p < 0.01) (Fig. 1F–H). These results suggested that lysosomes mediated CCl_4_-induced hepatocyte injury.

### 58-F Attenuates H_2_O_2-_induced Cell Viability, Death, and Restores the Inhibition of Proliferation

**Figure 2:**
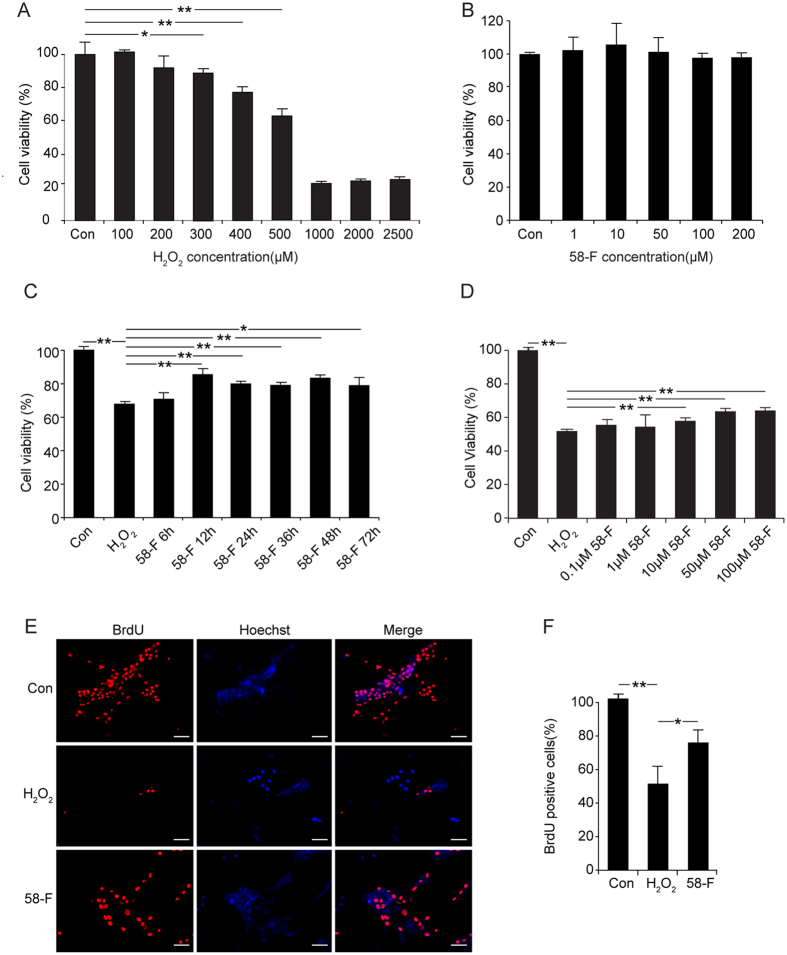
Effect of 58-F on cell viability and proliferation. (**A**) The effects of different concentrations (0–2500 μM) of H_2_O_2_ on cell viability were determined. Four thousand cells seeded in 96-well plates were treated with H_2_O_2_ for 2 h. (**B)** The effects of different concentrations (1–200 μM) of 58-F on cell viability were determined. Four thousand cells seeded in 96-well plates were treated with 58-F for 24 h (**C**) The effects of 58-F on changes in cell viability induced by H_2_O_2_ at different time intervals were measured. Four thousand cells in 96-well plates were treated with 50 μM 58-F, and 500 μM of H_2_O_2_ was added 2 h before the end of incubation. (**D**) The effect of different concentrations (0.1–100 μM) of 58-F on changes in cell viability induced by H_2_O_2_ were measured. (**E,F**) For the BrdU incorporation assay, 4000 cells seeded in a 96-well plate were treated with 50 μM of 58-F for 24 h, and with 500 μM H_2_O_2_ for an additional 8 h. A final concentration of 10 μM 5-bromo-2-deoxyuridine was supplemented in the medium for 24 h (scale bar = 50 μm). All data are presented as the mean ± SD. Statistical analysis was performed using Student’s *t* test. (*p < 0.05, **p < 0.01).

Cell viability under various concentrations of H_2_O_2_ (0, 100, 200, 300, 400, 500, 1000, 2000, and 2500 μM) is illustrated in Fig. 2A. The cell viability was inhibited by H_2_O_2_ at concentrations in the range of 300 μM∼500 μM, with a number of dead cells detected at concentrations in the range of 1000 μM∼2500 μM. The inhibition rate of 500 μM H_2_O_2_ was approximately 30∼50% (Fig. 2C,D). The cells were exposed to 58-F at concentrations of 0, 1, 10, 50,100 or 200 μM for 24 h. The results revealed that 58-F concentrations from 1 μM to 200 μM were not cytotoxic to hepatocytes (Fig. 2B). The cells were effectively protected by 58-F at different concentrations ranging from 10 μM to 100 μM. The time-course for the effectiveness of 58-F (50 μM) ranged from 6 h to 72 h of pretreatment before the addition of 500 μM H_2_O_2_ for 2 h (Fig. 2C,D). These results suggested that the 58-F compound protected hepatocytes from H_2_O_2_ injury in a dose- and time-dependent manner.

Instead of thymidine, BrdU was incorporated into the nuclear DNA during the S-phase of the cell cycle to evaluate cell proliferation^14^. After exposure to H_2_O_2_, the number of BrdU-positive cells was significantly decreased (49.6% of the control). However, pretreatment with 50 μM of 58-F significantly increased the number of BrdU-positive cells (74.3% of the control) (Fig. 2E,F).

Annexin V is a Ca^2+^-dependent phospholipid-binding protein with a high affinity for externalized phosphatidylserine, which is strictly confined to the inner leaflet of the plasma membrane facing the cytosol in viable cells. Localization of Annexin V from the inner to the outer plasma membrane is an early feature of apoptosis that occurs prior to the loss of membrane integrity^15^. Furthermore, living cells excluding PI allow for the specific detection and quantification of apoptosis.

**Figure 3:**
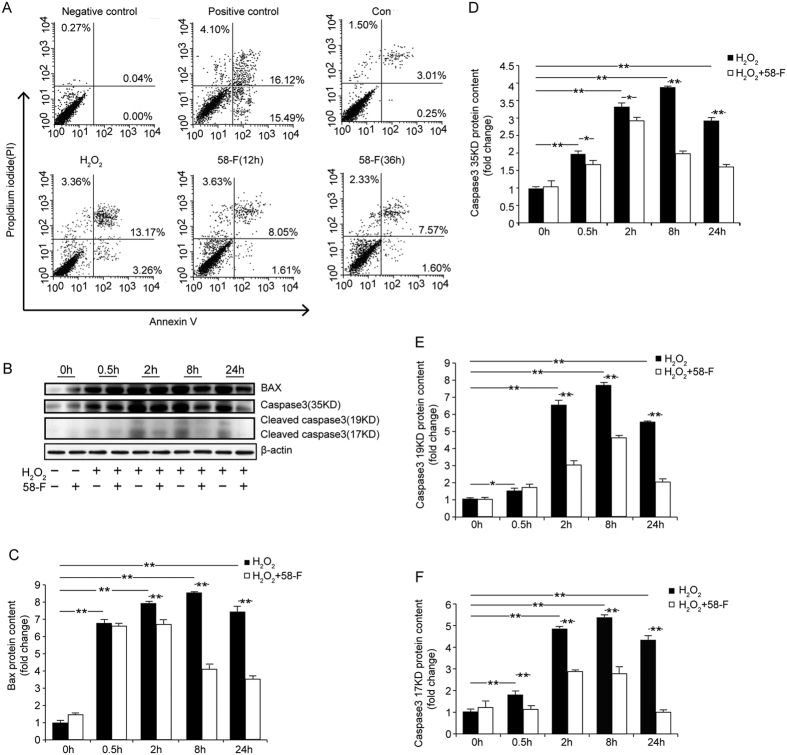
Effect of 58-F on cell death. (**A**) For the Annexin V/PI assay with FACS, the cells were treated with 50 μM of 58-F for 12 h or 36 h followed by an additional 2 h with 500 μM H_2_O_2_. (**B–F**) Bax and caspase3 protein levels were examined by Western blotting and were quantified. All data are presented as the mean ± SD. Statistical analysis was performed using Student’s *t* test. (*p < 0.05, **p < 0.01).

As shown in Fig. 3A, in the untreated control, only 0.25% of the cells were early apoptotic cells, 3.01% of the cells were late apoptotic cells and 1.50% were dead cells. By contrast, in the H_2_O_2_-treated group, 3.26% of the cells were early apoptotic cells, 13.17% of the cells were late apoptotic cells and 3.36% were dead cells, suggesting an approximately four-fold increase in the number of late apoptotic or dead cells in the H_2_O_2_-treated group compared with the untreated controls.

Following 12 h of treatment with 50 μM 58-F and 500 μM H_2_O_2_, 1.61% of the cells were early apoptotic cells and 8.05% of the cells were late apoptotic cells. At the 36 h time point, 1.60% of the cells were early apoptotic cells and 7.57% of the cells were late apoptotic cells (Fig. 3A). These results demonstrated that 58-F protected hepatocytes from H_2_O_2_-induced apoptosis and death.

The pro-apoptotic protein Bax has a carboxy-terminal α-helix domain of the membrane-anchoring region, which is normally sequestered in an inhibitory hydrophobic groove of Bax, preventing its insertion into membranes. Upon exposure to various death stimuli, the conformation of Bax is altered, and its membrane-anchoring domain is exposed and inserted into membranes, triggering apoptosis^16^. Our results showed that after exposure to H_2_O_2_, Bax protein expression increased gradually, peaked at 8 h, and subsequently decreased at 24 h. The levels of Bax in the 58-F treated groups were less than in the H_2_O_2_ group at the same time point (Fig. 3B,C).

Caspase-3 plays a central role in cell death pathways. Procaspase-3 exists in cells as inactive dimers. Upon activation, procaspase-3 separates into a large and a small subunit. The active caspase-3 upregulates the downstream enzymes of the caspase family and ultimately contributes to cell death^17^. In the present study, either procaspase-3 or cleaved active caspase-3 protein levels increased and peaked at 8 h and declined at 24 h after treatment with H_2_O_2_. Treatment with 58-F significantly suppressed the increase at the same time points (Fig. 3B,D,E,F). In summary, H_2_O_2_ induced both apoptotic and necrotic cell death and, therefore, inhibited cell proliferation and viability. The compound 58-F protected hepatocytes from death and promoted their proliferation.

### H_2_O_2_ Induces Oxidative Damage and 58-F Exhibits an Anti-oxidative Effect

**Figure 4:**
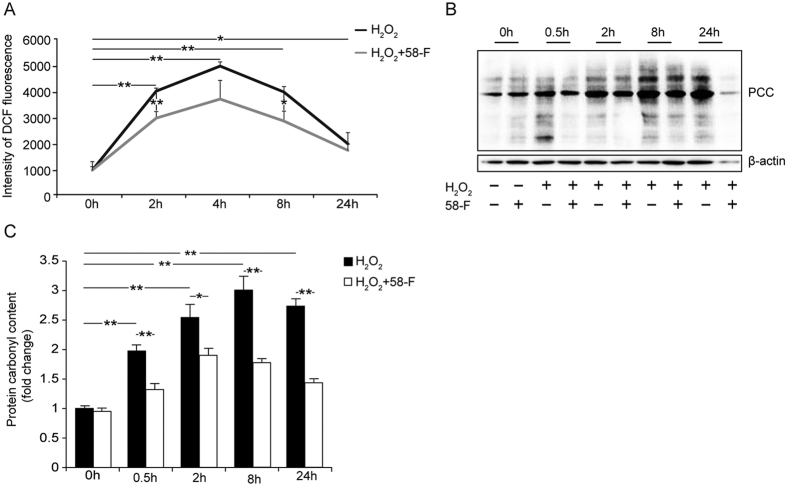
Effect of 58-F on oxidative damage induced by H_2_O_2_. (**A**) The ROS contents in cells were determined. After treatment with 58-F and H_2_O_2_, the cells were incubated with 10 mM H_2_DCF-DA at 37 °C for 15 min. (**B,C**) The PCC in cells was assayed by Western blotting and quantified. All data are presented as the mean ± SD. Statistical analysis was performed using Student’s *t* test. (*p < 0.05, **p < 0.01).

Progressive H_2_O_2_ treatment elevated the ROS content gradually. The levels peaked at 4 h and then decreased gradually to nearly normal levels at 24 h. The ROS content in the cells pretreated with 50 μM 58-F was significantly reduced compared with the cells receiving a single H_2_O_2_ treatment from 2 h to 8 h (Fig. 4A).

Proteins are the major target for ROS because amino acids are susceptible to oxidation. Carbonylation is the most common oxidative alteration of proteins. The results shown in Fig. 4B,C demonstrate that the amount of PCC increased progressively from 0.5 h to 24 h, although it peaked at 8 h and remained at a high level at 24 h. Similar results were obtained *in vivo* (Fig. 1D,E).

### H_2_O_2_ Induces LMP and 58-F Protects the Lysosome Membrane

**Figure 5:**
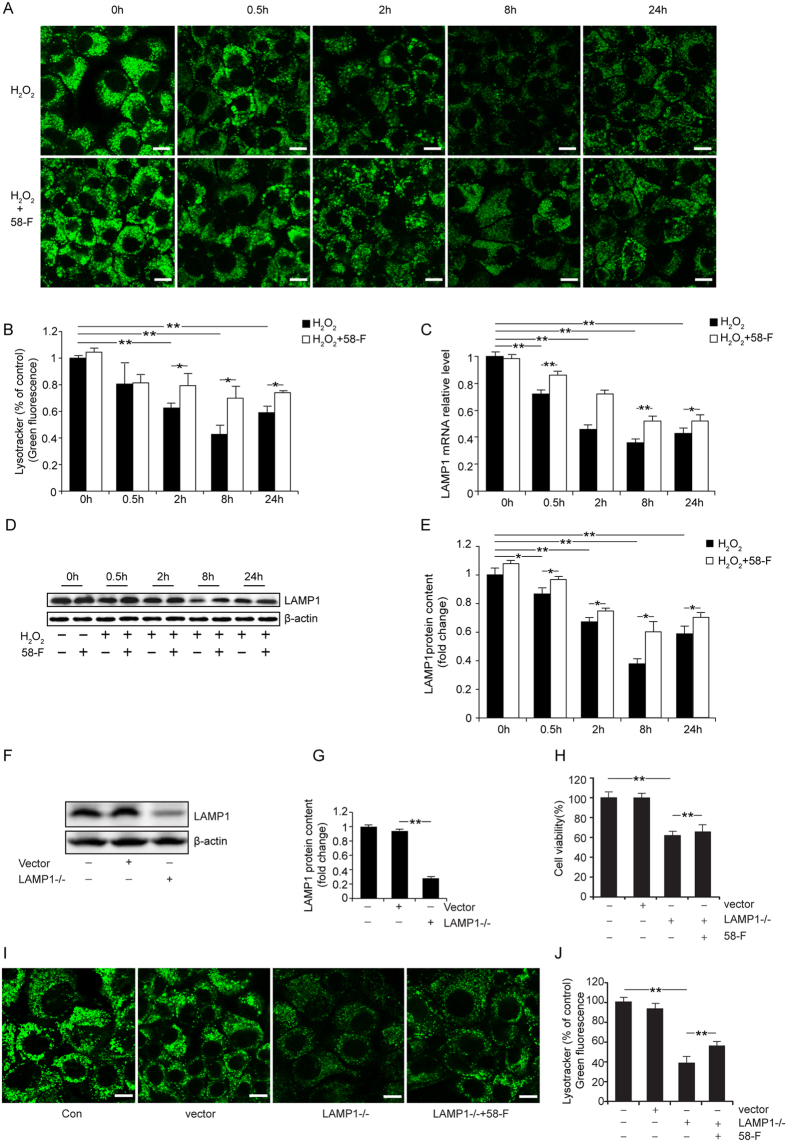
Effect of 58-F on lysosomal membrane permeability. (**A**) LysoTracker Green staining (scale bar = 10 μm) is shown. Ten thousand cells were plated in a 60 mm confocal plate, and after the designated treatments, the cells were incubated with 75 nM LysoTracker Green for 30 min at 37 °C. (**B**) Quantification of the LysoTracker Green staining is shown. (**C**) Levels of LAMP1 mRNA were measured in cells by qRT-PCR. (**D,E**) Levels of LAMP1 protein were measured in cells by Western blotting and quantification is shown. (**F,G**) LAMP1 shRNA inhibition efficiency was determined by Western blot analysis, and quantification is shown. (**H**) The cell viability of LAMP1 shRNA cells was determined. (**I,J**) LysoTracker Green staining of LAMP1 shRNA cells (scale bar = 10 μm) is shown. All data are presented as the mean ± SD. Statistical analysis was performed using Student’s *t* test. (*p < 0.05, **p < 0.01).

LysoTracker Green, a lysosomal marker that is selectively retained in the partially acidic lysosome and reflects the lysosomal membrane permeability^18^, was used to evaluate the integrity of the lysosome membrane. The intensity of green fluorescence gradually decreased with the increasing duration of H_2_O_2_ treatment and reached the lowest point at 8 h. It then increased slightly at 24 h. However, the fluorescent intensity increased with the 58-F pretreatment (Fig. 5A,B).

LAMP1 is highly expressed in lysosomes. Only 1–2% of the total LAMP1 can be found at the plasma membrane; its primary localization seems to be at the endosome-lysosomal membrane^18^. Our results reveal that LAMP1 protein levels gradually decreased with the increasing duration of H_2_O_2_ treatment, reaching its lowest level at 8 h, with a partial restoration of LAMP1 levels observed at 24 h. By contrast, in cells pretreated with 58-F, the LAMP1 protein levels significantly increased following a similar time course as the H_2_O_2_ treatment (Fig. 5D,E). Again, changes in LAMP1 mRNA expression were consistent with the levels of protein expression (Fig. 5C). The knockdown of LAMP1 expression in cells strongly diminished the intensity of green fluorescence in the lysosome (Fig. 5I,J) but also inhibited the cell viability (Fig. 5H). These data indicate that a deficiency in LAMP1 directly inhibits cell viability. Furthermore, in LAMP1 knockdown cells, the compound 58-F enhanced the fluorescence intensity of the LysoTracker Green and increased cell viability (Fig. 5H–J).

### 58-F Antagonizes H_2_O_2_-induced CatB/D and Proton Leakage

**Figure 6:**
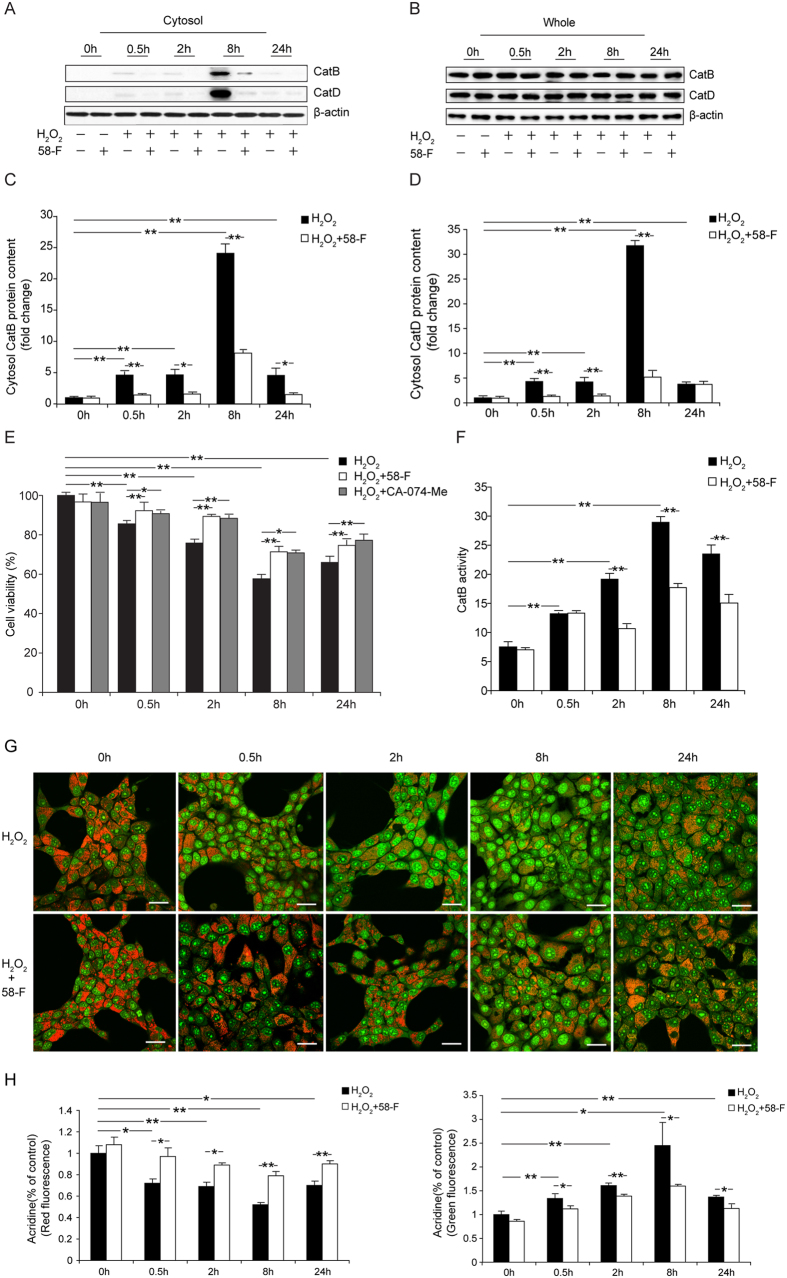
Effect of 58-F on the leakage of CatB/D into the cytosol. (**A**–**D**) Cat B/D levels were measured in the cytosol/whole lysate by Western blotting, and the quantification is shown. (**E**) Cell viability with 50 μM CA-074-Me (CatB inhibitor) treatment was determined. (**F**) CatB activity analysis was performed with chemiluminescence detection. (**G**) AO staining (scale bar = 25 μm) results are shown. After the designated treatments, cells seeded in a 60 mm confocal plate were incubated with 5 mg/ml AO for 30 min at 37 °C. All data are presented as the mean ± SD. Statistical analysis was performed using Student’s *t* test. (*p < 0.05, **p < 0.01). (**H**) Quantification of the AO staining is shown.

To further investigate whether catalytic enzymes in the lysosome leaked into the cytosol and caused the inhibition of cell viability, we assessed both the content and the activity of cathepsin B (CatB) and D (CatD) in the cytosol and in whole cells. Surprisingly, the content of both CatB and CatD in the cytosol were extremely high after exposure to H_**2**_O_**2**_ for 8 h, reaching increases of ∼24-fold for CatB and ∼31-fold for CatD (Fig. 6A,C,D). By contrast, the content detected in the whole cells remained unchanged (Fig. 6B). The compound 58-F suppressed cytoplasmic CatB and CatD content at all of the designated time points in a time-dependent manner (Fig. 6A,C,D).

The CatB activity in the cytosol gradually increased with the increased duration of H_2_O_2_ treatment, peaking at 8 h, and then declining at 24 h (Fig. 6F). The compound 58-F suppressed cytoplasmic CatB activity at all of the designated time points (Fig. 6F).

To further analyse whether the enzymatic activity of CatB in the cytosol induced cell death, the Cat B inhibitor CA-074-Me^19^ was used. As expected and consistent with the results of compound 58-F, CA-074-Me blocked the H_2_O_2-_induced cell death in a time-dependent manner (Fig. 6E).

AO is a cell-permeable fluorescent dye with a concentration-dependent fluorescence emission that ranges from red (when the concentration is high in the lysozyme) to green (when the concentration is low in the cytosol). AO is positively charged at a low pH, which consequently hinders the ability of the AO molecules to cross the vesicular membrane and escape into the surrounding cytoplasm. In the case of LMP, AO is released from the lysosomes into the cytosol where it emits an enhanced green fluorescence that can be monitored by fluorescence microscopy^20^. After delivery of H_2_O_2_ into the cells, a significant reduction of red fluorescence and a concomitant increase in green fluorescence was observed in a time-dependent manner, suggesting that H_2_O_2_ induced LMP at the cellular level (Fig. 6G,H). By contrast, the compound 58-F was associated with an increase of the red fluorescence and a concomitant suppression of the green fluorescence at all of the designated time points compared with those in the H_2_O_2_-treated cells (Fig. 6G,H).

### Lysosomal acidification inhibition by H_2_O_2_ Contributes to Cell Death

**Figure 7:**
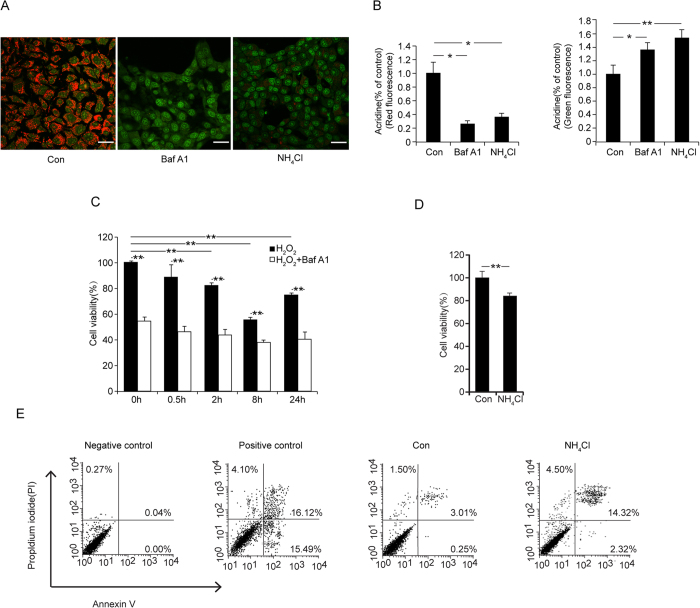
lysosomal acidification inhibition leads to cell death. (**A**) AO staining (scale bar = 25 μm) results are shown, the cells were treated with 100 nM BafA1 for 24 h or 100 μM NH_4_Cl for 2 h. All data are presented as the mean ± SD. Statistical analysis was performed using Student’s *t* test. (*p < 0.05, **p < 0.01). (**B**) Quantification of the AO staining is shown. (**C**) Cell viability: The cells seeded in 96-well plates were incubated with 100 nM of BafA1(V-ATPase inhibitor) for 24 h followed by H_2_O_2_ for 0.5 h, 2 h, 8 h and 24 h. (**D**) Cell viability: Cells seeded in 96-well plates were incubated with 100 μM of NH_4_Cl for 40 min. All data are presented as the mean ± SD. Statistical analysis was performed using Student’s *t* test. (**p < 0.01). (**E**) For the Annexin V/PI assay with FACS, the cells were treated with 100 μM NH_4_Cl for 2 h.

The vacuolar H^+^-ATPases (V-ATPases) within the lysosomal membranes are responsible for establishing and maintaining a low pH. Bafilomycin A1 (BafA1) specifically inhibits the V-ATPase by binding to its proteolipid subunit and blocking the passage of protons into the lysosome, and therefore hinders lysosomal acidification^21^ (Fig 7A,B). The results show that cell viability was inhibited after H_2_O_2_ treatment and progressively suppressed by BafA1 at all of the designated time points (Fig. 7C). In addition, NH_4_Cl was in equilibrium with the weak base NH_3_, which is membrane permeable and enters acidic compartments to bind with protons, and therefore, it was added to artificially acidify the cytosol and alkalify the lysosome^22^ (Fig. 7A,B). Similar cell viability results were found in cells treated with NH_4_Cl (Fig. 7D). Furthermore, we observed that 2.32% of cells were early apoptotic,14.32% of cells were late apoptotic and 4.50% were dead following NH_4_Cl treatment compared with control groups, in which 0.25% were late apoptotic, 3.01% were late apoptotic and 1.50% were dead cells (Fig. 7E). Together, the LMP caused by H_2_O_2_ leads to lysosomal acidification inhibition and contributes to cell death.

### N-acetylcysteine (NAC) is Protective against Cellular Toxicity by Inhibiting LMP

**Figure 8:**
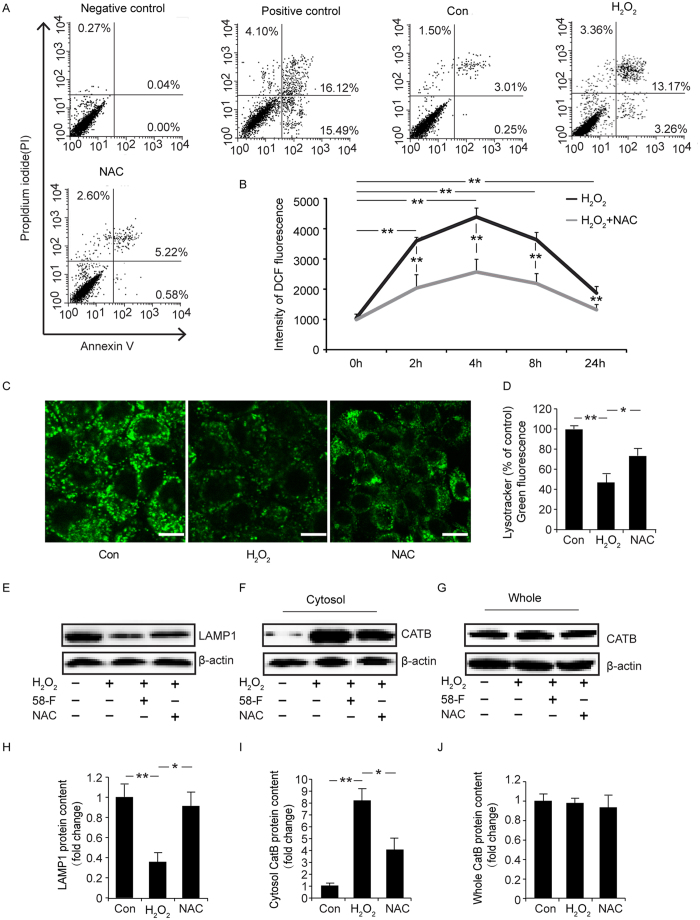
Effect of NAC on ROS content, cell death , lysosomal membrane permeability and the leakage of CatB to the cytosol. To identify the effect of NAC *in vitro*, cells were treated with 100 μg/ml of NAC for 12 h followed by an additional 2 h with 500 μM H_2_O_2_ for the assay with FACS. For the ROS content assay, the cells were treated with 100 μg/ml NAC for 24 h followed by 500 μM H_2_O_2_ for different amounts of time. In other assays, the cells were treated with 100 μg/ml NAC for 16 h followed by an additional 8 h with 500 μM H_2_O_2_. (**A**) The Annexin V/PI assay with FACS is shown. (**B**) The ROS contents in cells is shown. (**C**) LysoTracker Green staining (scale bar = 10 μm) is shown. (**D**) Quantification of the LysoTracker Green staining is shown. (**E,H**) Levels of LAMP1 protein were measured in cells by Western blotting and quantification is shown. **(F**,**G,I,J**) Cat B/D levels were measured in the cytosol/whole lysate by Western blotting, and the quantification is shown. (*p < 0.05, **p < 0.01).

ROS production in cells was detected using 2′,7′–dichlorofluorescein diacetate (DCFH-DA), as described in the Materials and Methods, and was decreased in the presence of NAC pretreatment (Fig. 8B). In addition, LAMP1 protein levels in the cells that received NAC pretreatment were significantly increased compared with the cells that received the H_2_O_2_ treatment. The content of CatB in the cytosol following NAC treatment also showed a significant decrease compared with that in the H_2_O_2_ treatment group. Again, the LysoTracker Green stain was used to evaluate the integrity of the lysosome membrane, and cells treated with NAC showed an increase in the intensity of fluorescence.

As shown in Fig. 8A, after treatment with 100 μg/ml NAC after 500 μM of H_2_O_2_, 3.26% of the cells were early apoptotic and 13.17% of the cells were late apoptotic after 12 h, whereas in the H_2_O_2_ treatment group, 0.58% of the cells were early apoptotic and 5.22% of the cells were late apoptotic(Fig. 8A). Together, these results showed that NAC protected hepatocytes from H_2_O_2_-induced apoptosis and death by inhibiting LMP.

## Discussion

### Hepatocyte death mediated by CCl_4_-induced lysosome injury

CCl_4_ is a potent hepatotoxin that is widely used for the induction of chemical liver damage. The toxicity of CCl_4_ is attributed to accumulation of ROS during its metabolism, which is involved in the pathological progress of liver diseases. Therefore, oxidative stress is one of the main mechanisms of CCl_4_ -induced liver injury^23^. Our *in vivo* studies suggest that the levels of lipid peroxidation, protein oxidation, and serum ALT activity in hepatocytes following cell injury or death are increased after CCl_4_ treatment. A number of studies have suggested that oxidized proteins that are not cleared in time lead to the loss of cell viability and persistent cell death^24^. The livers of CCl_4_-induced mice also showed large areas of dead hepatocytes. The molecular structure of LAMP1 is composed of mainly carbohydrates that form a nearly continuous coating on the inner surface of the lysosome membrane and serve as a barrier to soluble hydrolases, preventing the release of lysosomal enzymes and H^+^ into the cytoplasm^25^. The loss of LAMP1 leads to damage to the integrity of the lysosome membrane and ultimately to the leakage of the lysosome contents. The mRNA and protein levels of LAMP1 detected in the liver were lower after CCl_4_ treatment than under normal conditions, which suggested that lysosomes mediated the CCl_4_-induced hepatocyte injury/death that induced injury to the entire liver.

### Following cathepsin leakage and lysosomal acidification inhibition, LMP triggers lysosomal cell death in H_2_O_2_-induced hepatocyte injury

H_2_O_2_-induced cytotoxicity is a common method used for measuring potential antioxidants. In this study, treatments with different concentrations (0–2500 μM) of H_2_O_2_ for 2 h induced a dose-dependent decrease in cell viability. The rate of inhibition achieved by 500 μM H_2_O_2_ was approximately 40%; thus, this concentration was used for the subsequent experiments. The ROS products increased gradually, peaking at 4 h, in tandem with the protein carbonyl content which peaked at 8 h. Following the oxidative damage, cell viability was inhibited, and cell proliferation was diminished, whereas apoptotic and necrotic cell death increased.

In cultured cells, LMP occurred following H_2_O_2_ injury^26^,^27^. The LMP followed by the leakage of enzymes, including CatB, triggers lysosome-induced cell death^28^, and hepatocyte death is accompanied by an increase in LMP in acute liver injury^29^. According to the cell type and the dosages of the inducing factors, lysosomal apoptosis or necrosis occurs^30^. In our study, both LysoTracker and AO staining showed that the LMP increased gradually with time and was restored after peaking at 8 h.

LAMP1 is a highly glycosylated protein located in the lysosomal membrane that controls the integrity of the lysosomal membrane^25^. Our studies found that the mRNA and protein levels of LAMP1 decreased after hepatocyte injury *in vitro* and *in vivo.* Furthermore, after knockdown of LAMP1 in hepatocytes, LMP occurred and cell viability was inhibited, which demonstrated that LMP mediated CCl_4_/H_2_O_2_-induced hepatocyte injury/death.

Under physiological conditions, proteolytic, and hydrolytic enzymes in the lysosomes were restricted to the intact lysosomal membranes. Upon LMP, H^+^ was released into the cytoplasm resulting in acidification and cellular apoptosis^31^,^32^. NH_4_Cl and Bafilomycin A1 were used to evaluate the effects of lysosomal acidification inhibition on cell viability and death, respectively. The results indicated that Bafilomycin A1 further inhibited cell viability after cells were exposed to H_2_O_2_ at all of the designated time points. Furthermore, the results of the NH_4_Cl treatment were consistent with that of Bafilomycin A1 and resulted in apoptosis.

In LMP, hydrolytic enzymes in the lysosomes are released into the cytoplasm^33^,^34^. Cathepsins, including cathepsin B/D/L, under near-neutral pH conditions also exhibited catalytic activity for a few hours^35^. CatB leakage from the lysosomes is also associated with apoptosis^36^,^37^. Our studies found that levels of CatB and D in the cytoplasm highly increased after exposure to H_2_O_2_ for 8 h, whereas the overall cellular levels remained unchanged. Furthermore, the CatB activity in the cytoplasm increased over time following the same time course as the H_2_O_2_ treatment. Moreover, the CatB-specific inhibitor, CA-074-Me increased the cell viability in the presence of H_2_O_2_. Therefore, our results confirmed that upon LMP, protons and lysosomal enzymes, including CatB/D, were translocated to the cytoplasm and triggered cell death.

### Effect of 58-F on hepatocyte injury/death

The compound 58-F is a flavanone that has been newly extracted from *Ophiopogon japonicus*, with no published reports of its pharmacological activity with regard to hepatic protection. Here, we evaluated its effect on hepatocyte protection. The *in vivo* results revealed that 58-F decreased serum ALT activity, scavenged products of lipid peroxidation and oxidized proteins, and restored LAMP1 levels, suggesting that 58-F is a potentially new protective agent for hepatocytes. H_2_O_2_-induced hepatocyte damage was used to evaluate the mechanisms of 58-F on hepatocyte protection *in vitro*. 58-F reduced the apoptotic and dead cell numbers and prevented the inhibition of cell proliferation induced by H_2_O_2_ by clearing the oxidized products of lipids and proteins, also having protective effects on hepatocytes *in vitro.* A large amount of H_2_O_2_ diffused into the lysosome resulting in LMP^38^. In wild-type astrocytes, H_2_O_2_-induced expression of LAMP1 decreased and lysosomal LMP was decreased^39^. (-)-Epigallocatechin-3- gallate (EGCG) promotes the production of intracellular ROS upstream of LMP and cell death, and the antioxidant NAC protects against EGCG-mediated LMP by decreasing the level of ROS^40^. In addition, in Bxpc3 cells, LAMP1-shRNA significantly reduced the viability of cells and LysoTracker stain. Substantial amounts of ROS were detected with H_2_O_2_ treatment, and these levels were decreased by NAC^41^. In this study, we also found that H_2_O_2_ induced LMP by decreasing LAMP1 expression and NAC. Similarly, 58-F stabilized the integrity of the lysosomal membrane partly by upregulating the expression of LAMP1, following the diminishing proton and cathepsin leakage into cytoplasm.

In brief, the compound 58-F protected the integrity of the lysosomal membrane by upregulating the expression of LAMP1, which reduced cathepsin B/D and H^+^ leakage into the cytosol, and protected hepatocytes from death.

## Methods

### 58-F compound

**Figure 9:**
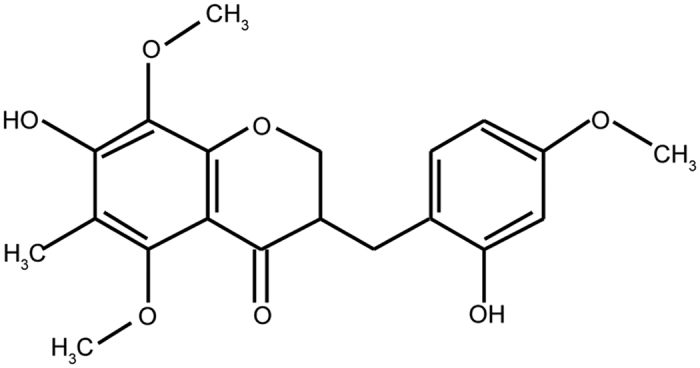
Molecular Structure of 58-F. This copyrighted image is used with permission from Xi Gong, affiliated with the Shanghai Yi-Lin biotechnology limited corporation.

58-F (molecular weight: 374.38; molecular formula: C20H22O7; CAS: 4477336-79-1; (Fig. 9) was purchased from the Shanghai Yi-Lin biotechnology limited corporation (Yi-Lin, Shanghai, China) and was dissolved in 0.1% dimethyl sulphoxide (DMSO)-Dulbecco’s modified eagle medium (DMEM) (Hyclone, Waltham, MA, USA) supplemented with 2% foetal bovine serum (GIBCO, Waltham, MA, USA).

### Animal experiments

In total, 24 male BALB/c mice (license number: SCXK (Shanghai) 2012-0002) were housed at the animal centre of the Shanghai University of Traditional Chinese Medicine and fed a standard pelleted diet and water. All of the experimental protocols were approved by the Shanghai University of Traditional Chinese Medicine’s Animal Ethics Committee, and all of the methods in the study were performed in accordance with the approved guidelines.

The mice were initially randomized into three groups: a control group (n = 6), a CCl_4_-treated group (n = 9) and a 58-F/CCl_4_-treated group (n = 9). Before CCl_4_ injection, the mice in the control and CCl_4_ groups were injected intragastrically with 0.9% NaCl. The mice in the 58-F/CCl_4_-treated group were intragastrically administered 58-F at a dosage of 15 mg/kg body weight for 3 days. The mice in the CCl_4_-treated and 58-F/CCl_4_-treated group were given intraperitoneal injections of 5% CCl_4_ (10 μl/g weight), and the mice in the control group were intraperitoneally injected with olive oil (10 μl/g weight). All of the mice were sacrificed on day 7 of the experiment. Sera were collected for the alanine aminotransferase (ALT) activity assay according to the recommendations of the Institute of Biological Products Nanjing Jiancheng (Nanjing, China). The tissues were collected for malonaldehyde (MDA) assay according to the manufacturer’s instructions (Cayman, Ann Arbor, MI, USA).

### H&E staining

The 10% neutral formaldehyde fixed liver tissues were processed in an automated tissue processor that has been optimized for liver tissues, embedded in paraffin blocks and cut into 4-μm thick sections. The sections were stained with H&E, followed by visualization of the histopathological changes under a light microscope.

### Cell Culture and Short-hairpin RNA (shRNA) Transduction

The BNL CL.2 mouse embryonic hepatocyte cell line was purchased from the cell bank of the Shanghai Institutes for Biological Sciences and was routinely grown in DMEM supplemented with 10% FBS, 100 U/ml penicillin and 100 μg/ml streptomycin.

The BNL CL.2 mouse embryonic hepatocyte cells were incubated with the LAMP1-specific shRNA (target sequences: 5′-GCCACTGTGGGAAACTCATAC-3′) and lentiviral particles (pHBLV-U6-ZsGreen-Puro, Hanbio Biotechnology Co., Ltd., No 1 Building, 150 Cailun Road, Pudong New Area, Shanghai, China) at a ratio of 2 particles to 1 cell. After 48 h, the medium was replaced with 1.5 μg/ml puromycin to improve the transduction efficiency. Successfully transduced cells included a puromycin resistance tag, and these cells were selected using 1.5 μg/ml puromycin. LAMP1 knockdown was confirmed using quantitative RT-PCR and Western blot analysis.

All of the cells were cultured in a humidified atmosphere containing 5% CO_2_ at 37 °C, unless otherwise indicated.

### Cell viability assay

Cell viability was assessed by a Cell Counting Kit-8 (CCK8) assay (Dojindo, Kumamoto, Japan): 4000 cells were seeded in 96-well plates and were incubated for 24 h at 37 °C in 5% CO_2_. After incubation with different concentrations of 58-F, 100 μM ammonium chloride (NH_4_Cl) or 50 μM CA-074-me and 10 μl CCK8 reagent was added to each well and incubated for 2 h at 37 °C. The absorbance at 450 nm was measured using a microplate reader (Bio-Tek, Hercules, CA, USA). The mean absorbance values from 4 wells following each treatment were used to represent the index of cell viability. The experiments were repeated at least in triplicate. The cell viability was expressed as a percentage of the control level.

### Detection of ROS by DCFH-DA

Intracellular ROS levels were assessed using 2′,7′–dichlorofluorescein diacetate (DCFH-DA). DCFH-DA is a stable, fluorogenic and non-polar compound that readily diffuses into the cells. It is deacetylated by intracellular esterases to the non-fluorescent 2′,7′-dichlorodihydrofluorescein (DCFH), which is later oxidized by intracellular ROS into the highly fluorescent 2′,7′-dichlorofluorescein (DCF)^42^. The intensity of fluorescence is proportional to the intracellular ROS levels. The cells were incubated with 10 mM H_2_DCF-DA at 37 °C for 15 min. After incubation, the cells were washed. DCF fluorescence intensity was measured in an automatic microplate reader (Synergy 2; BioTek, Vermont, USA) at an excitation wavelength of 485 nm and emission wavelength of 535 nm.

### BrdU labelling and immunofluorescence

After growing 4000 cells in a 96-well plate treated with 50 μM 58-F for 24 h, and with 500 μM H_2_O_2_ for an additional 8 h, a final concentration of 10 μM 5-bromo-2-deoxyuridine (BrdU, Sigma, St Louis, MO, USA) was supplemented in the medium for 24 h at 37 °C. The cells were fixed with cold 4% paraformaldehyde for 15 min at room temperature. After washing with phosphate buffer saline (PBS), cells were incubated with 0.2% TritonX-100 for 20 min to permeabilize the membranes. The BrdU epitope was exposed by incubating the cells with 2 N HCl for 0.5 h, at 37 °C. After washing with PBS, cultures were blocked with 10% horse serum in PBS. A primary antibody (CST, Boston, MA, USA) was added at a 1:100 dilution in blocking solution, and incubated at 4 °C overnight. The cells were washed three times with PBS and incubated with a secondary antibody (AlexaTM594 donkey anti-mouse, Jackson ImmunoResearch, West Grove, PA, USA) at a 1:100 dilution for 1 h at room temperature in a blocking solution. The nuclei were labelled with 5 μg/ml Hoechst 33342 (CST, Boston, MA, USA) for 1 h at 37 °C. BrdU-positive proliferating cells were expressed as a percentage of the control level.

### Annexin V-FITC/PI staining with fluorescence-activated cell sorter (FACS)

Cells treated with 50 μM 58-F for 12 h or 36 h following further 2 h treatment with 500 μM H_2_O_2_, or with 100 μM NH_4_Cl for 2 h were harvested through trypsinization, were washed twice with cold PBS three times and were resuspended in 100 μl of binding buffer at a density of 1.2 × l0^6^ cells per ml. Then, 5 μl of Annexin V-FITC (BD Pharmingen, Santiago, CA, USA) and 5 μl of propidium iodide (PI) (BD Pharmingen, Santiago, CA, USA) were added to each sample in the dark for 15 min. The fluorescence was detected immediately after staining using a flow cytometer (Ex = 488 nm; Em = 530 nm). The results were presented as the percentage of cells that were viable (Ann-V^−^ /PI^−^), early apoptotic (Ann-V^+^/PI^−^), late apoptotic (Ann-V^+^/PI^+^) or nonviable (Ann-V^−^/PI^+^). Cells treated with 1 μM staurosporine for 24 h was used as the positive control for apoptosis.

### Western blotting

At the end of the designated treatments, tissue homogenates and cell lysates were prepared in a buffer containing 62.5 mM Tris-HCl at pH 6.8, 20% glycerol, 2% SDS, 2 mM DTT, 100 mM PMSF and proteinase inhibitor cocktail. Protein concentrations were determined using the BCA total protein assay (Biomiga). Samples with equal amounts of protein were subjected to a 12% SDS-PAGE and transferred to a polyvinylidene difluoride (PVDF) membrane (Bedford, MA, USA). After blocking with 5% non-fat milk, the membrane was probed with the designated primary and secondary antibodies. The membranes were developed using enhanced chemiluminescence (Thermo Scientific, Waltham, MA, USA) and visualized using a FCM gel imaging device (Protein Simple, San Francisco, CA, USA).

### Protein carbonyl content

The protein carbonyl content (PCC) was assayed using an Oxyblot kit (Millipore, Bedford, MA, USA). Briefly, 5 μl of the tissue homogenates or cell lysates including 20 μg of protein mixed with 5 μl 12% SDS, were supplemented with 10 μl of 2,4-dinitrophenylhydrazine in 2N HCl to derivatize the carbonyl groups from the protein side chains for 15 min at room temperature. The derivatized samples were then separated using 12% SDS-PAGE. Western blotting was performed as described above, using the 2,4-dinitrophenylhydrazine antibody provided (1:50). Analyses of the results were reported as the ratio of the protein-of-interest/β-actin. Given the presence of multiple bands, the average value of all of the bands within each lane was used to provide an overall measure of protein carbonyl content.

### LysoTracker Green or Acridine Orange (AO) Staining

Ten thousand cells were plated in a 60 mm confocal plate. After the designated treatments, the cells were incubated with 75 nM LysoTracker Green (Invitrogen, Waltham, MA, USA) or 5 mg/ml AO (Sigma, St Louis, MO, USA) for 30 min at 37 °C. The fluorescence was examined using a confocal microscope with the excitation wavelength set at 488 nm. Two separate emission bands (505–570 nm and 615–754 nm) for AO staining and one for LysoTracker staining (504–511 nm) were used simultaneously, obtained and photographed.

### Cytosolic/particulate separation

The method for cytosolic/particulate separation was based on the protocol described in the Cytosol/Particulate Rapid Separation Kit (BioVision, Milpitas, CA, USA). Briefly, at the end of the designated treatments, both the medium and the adherent cells were harvested into 15 ml tubes, and the samples were centrifuged at 600 × g for 5 min. The pellets were resuspended in 40 μl of cell suspension buffer and mixed with 40 μl of cytosol releasing buffer, including 1 mM PMSF. The cell suspension was carefully layered upon 500 μl of an oil layer with 40 μl of a particulate layer at the bottom on ice for 30 s. After centrifugation at 1400 × g for 1 min, the cytosol was separated from the membrane particles by the oil layer and were transferred to new tubes. A BCA total protein assay kit (Biomiga) was used to determine the protein concentration.

### Real-time RT-PCR Analysis

Total RNA was isolated from the liver tissues (50 mg) or cells (1 × 10^6^) using the TRIzol reagent (Invitrogen, Waltham, MA, USA). Equal amounts of RNA were reverse transcribed into cDNA. The cDNA was amplified by real-time PCR, and the housekeeping gene 18S rRNA was used as an internal standard to quantify the levels of the target mRNA. The cycling conditions were as follows: hold: 95 °C for 10 s; cycling: 95 °C for 5 s, 60 °C for 30 s, 40 cycles; and melt for 65−95 °C. The nucleotide sequences for the primers used are as follows:

18 S rRNA: Forward: 5′-GTAACCCGTTGAACCCCATT-3′

Reverse: 5′-CCATCCAATCGGTAGTAGCG-3′

LAMP1: Forward: 5′-GATACAGTGGGGTTTGTGGG-3′

Reverse: 5′-CTGTCGAGTGGCAACTTCAG-3′

### Statistical Analysis

All data are presented as the mean ± SD. Statistical analysis was performed using Student’s t test and one-way ANOVA. Statistical significance was set at p < 0.05. At least three independent experiments were performed for each condition.

## Additional Information

**How to cite this article**: Yan, X. *et al*. 58-F, a flavanone from *Ophiopogon japonicus*, prevents hepatocyte death by decreasing lysosomal membrane permeability. *Sci. Rep.* **6**, 27875; doi: 10.1038/srep27875 (2016).
