# Author Correction: 58-F, a flavanone from *Ophiopogon japonicus*, prevents hepatocyte death by decreasing lysosomal membrane permeability

**DOI:** 10.1038/s41598-021-98198-z

**Published:** 2021-09-13

**Authors:** Xiaofeng Yan, Tingjie Ye, Xudong Hu, Pei Zhao, Xiaoling Wang

**Affiliations:** Department of Biology, School of Basic Medical Science, Shanghai University of Traditional Chinese Medicine, Shanghai, 201203 China

Correction to: *Scientific Reports* 10.1038/srep27875, published online 16 June 2016

This Article contains an error in Figure 8, where the treatments below the western blots in the panels E, F and G do not match the western blots. The correct Figure 8 and accompanying legend appear below.

**Figure 8:**
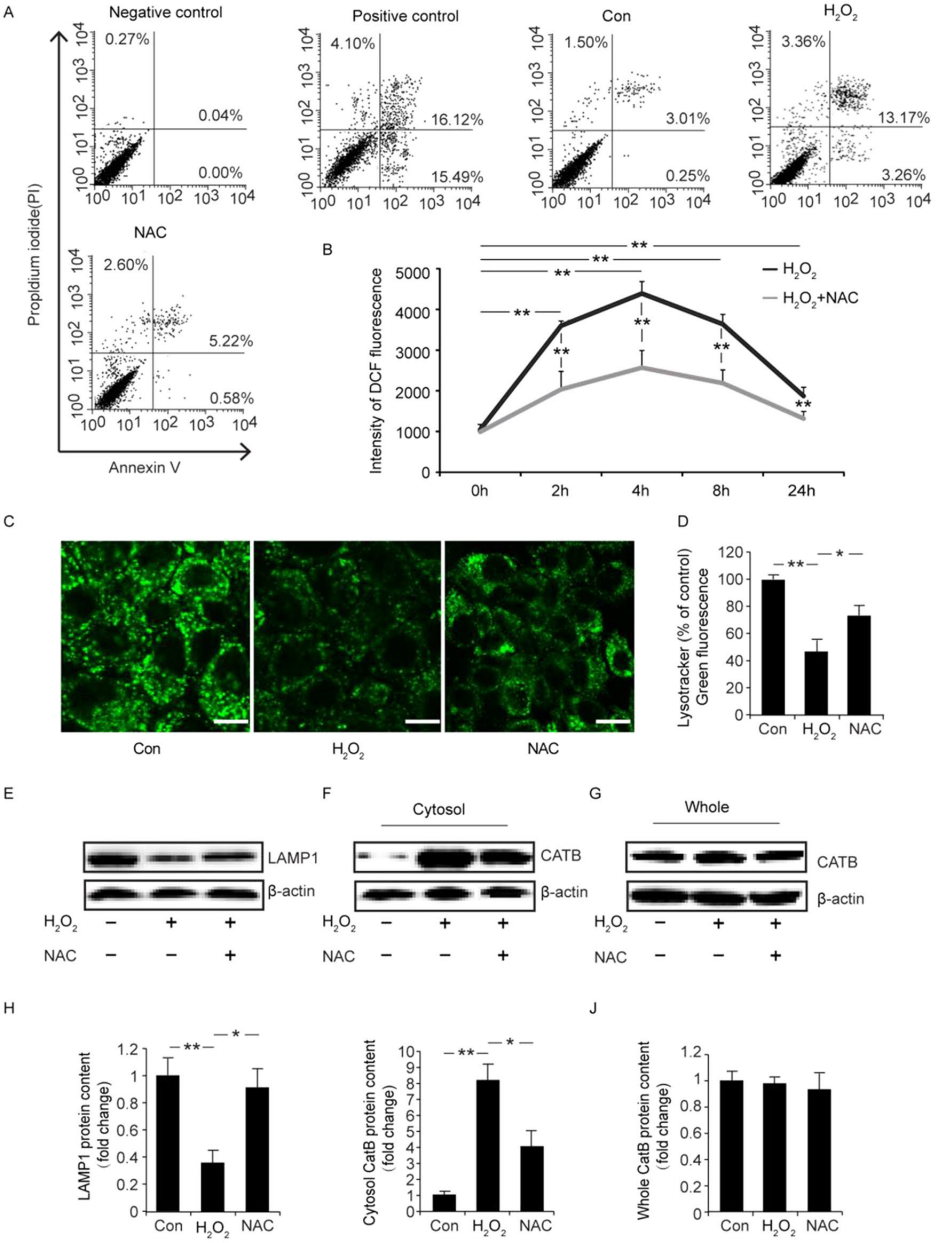
Effect of NAC on ROS content, cell death, lysosomal membrane permeability and the leakage of CatB to the cytosol. To identify the effect of NAC in vitro, cells were treated with 100 μg/ml of NAC for 12 h followed by an additional 2 h with 500 μM H_2_O_2_ for the assay with FACS. For the ROS content assay, the cells were treated with 100 μg/ml NAC for 24 h followed by 500 μM H_2_O_2_ for different amounts of time. In other assays, the cells were treated with 100 μg/ml NAC for 16 h followed by an additional 8 h with 500 μM H_2_O_2_. (**A**) The Annexin V/PI assay with FACS is shown. (**B**) The ROS contents in cells is shown. (**C**) LysoTracker Green staining (scale bar = 10 μm) is shown. (**D**) Quantification of the LysoTracker Green staining is shown. (**E, H**) Levels of LAMP1 protein were measured in cells by Western blotting and quantification is shown. (**F, G, I, J**) Cat B/D levels were measured in the cytosol/whole lysate by Western blotting and the quantification is shown (*p < 0.05, **p < 0.01).

